# The emerging role of brain neuroinflammatory responses in Alzheimer’s disease

**DOI:** 10.3389/fnagi.2024.1391517

**Published:** 2024-07-03

**Authors:** Mandana Amelimojarad, Melika Amelimojarad, Xiaonan Cui

**Affiliations:** Department of Oncology, The First Affiliated Hospital of Dalian Medical University, Dalian, China

**Keywords:** neuroinflammation, Alzheimer’s disease, microglia, NSAIDs, inflammation

## Abstract

As the most common cause of dementia, Alzheimer’s disease (AD) is characterized by neurodegeneration and synaptic loss with an increasing prevalence in the elderly. Increased inflammatory responses triggers brain cells to produce pro-inflammatory cytokines and accelerates the Aβ accumulation, tau protein hyper-phosphorylation leading to neurodegeneration. Therefore, in this paper, we discuss the current understanding of how inflammation affects brain activity to induce AD pathology, the inflammatory biomarkers and possible therapies that combat inflammation for AD.

## Background

Dementia is a noxious neurodegenerative disorder, and Alzheimer’s disease (AD), is the most common cause of dementia with increasing prevalence among the elderly (Aarsland, 2020). According to the World Health Organization (WHO), by 2050 the number of people with dementia will reach 132 million in the world (Porsteinsson et al., 2021; Ren et al., 2022).

AD is characterized by progressive neuropathological processes including cognitive function impairment and memory loss principally caused by increased accumulation of amyloid-β (Aβ) plaques, and hyperphosphorylated tau protein (Nakamura et al., 2018; Silva et al., 2019).

Different risk factors have been detected for AD development such as age, cardiovascular changes, metabolic disorders, increased metal ions accumulation, and brain injury (Baltes et al., 2011; Silva et al., 2019). Despite the vicious role of amyloid plaques and neurofibrillary tangles in the brain, the significant role of abnormal inflammation in inducing the inflammatory mediators release from brain cells, neurodegeneration, and loss of neuronal synapses is considered as the new hallmarks of AD pathology (Gouras et al., 2015; Newcombe et al., 2018; Muralidar et al., 2020).

Currently, nonsteroidal anti-inflammatory drugs (NSAIDs) and cholinesterase inhibitors are approved drugs to delay AD but none of them could cure the disease (Moride et al., 2003; Long and Holtzman, 2019). Therefore, identification of core pathologies mechanism responsible for AD, different proteins and genes associating with neuroinflammation and potential therapeutic targets is essential (Weggen et al., 2003; Benito-León et al., 2019).

In this review, we focused on an in-depth evaluation of the Blood–Brain Barrier (BBB), the brain cells especially the microglia modification in inducing the inflammatory responses as a new interest target of AD pathogenesis research. In addition, we highlighted all the inflammatory biomarkers with the potential to be used for targeted therapy.

## Brain cells connection with AD pathology

Microglia and astrocytes are the two main neuroglial cells, playing critical functions in Homeostasis, neuron development, differentiation, survival, synaptic plasticity, and neuronal metabolism (Zhang et al., 2021; McKee et al., 2023). The activation process of microglia and astrocytes is followed by a series of morphological and biological functions leading to the release of pro-inflammatory mediators and phagocytic activity (Weggen et al., 2003; Zhou and Hu, 2013; Greten and Grivennikov, 2019).

Microglia are one of macrophagic immune cells that reside in the central nervous system (CNS) and play important roles in surveillance and phagocytosis (Sheng et al., 2019). by recruiting other innate immune cells like neutrophils, dendritic cells, monocytes, invasive macrophages, and natural killer (NK) cells, Microglia are considered as important modulators of the innate immune response in the brain.

In response to infection, the inflammatory response activates resting microglia and encourages the release of free radicals (NO), reactive oxygen species (ROS), and pro-inflammatory cytokines (e.g., IL-1β, IL6, TNF). There are two types of activated microglia states: pro-inflammatory (M1-like; neurotoxic) and anti-inflammatory (M2-like; neuroprotective). Therefore, M1 and M2 polarization switches play the most significant role in the proper activation of microglia and release of pro-inflammatory mediators (Ransohoff, 2016; Greten and Grivennikov, 2019; Ho et al., 2020).

Although, Activation of microglia, seems to help in the clearance of Aβ during the chronic phase of neuroinflammation and early development of AD through phagocytosis (Villeda et al., 2011; Long and Holtzman, 2019). According to Newcombe et al. (2018), the pathogenesis of AD may be advanced by the microglia’s continuous brain stimulation in response to the accumulation of Aβ plaque, tau protein phosphorylation, and inflammatory responses which impairs their ability to phagocytose, produces pro-inflammatory mediators, and exacerbates tau and Aβ pathology (Leng and Edison, 2020).

Mutations in microglia-related genes have a substantial impact on the ability of microglia, causing them to become permanently activated, reducing their capacity for phagocytosis, and ultimately resulting in neuroinflammation and neurodegeneration (Zhang et al., 2021). Therefore, understanding the molecular mechanism of microglia is highly important to detect their dual roles in either Aβ plaques accumulation or degradation (Baltes et al., 2011).

Recently certain molecular regulators of microglial proliferation have been directly demonstrated to exist including triggering receptor expressed on myeloid cells 2 (TREM2) and apolipoprotein E (APOE) which are both among AD risk factors for Late Onset AD (LOAD) (Wolfe et al., 2018).

In the central nervous system, APOE plays multiple roles, such as maintaining lipid homeostasis, healing damaged neurons, eliminating toxins like Aβ, and immune responses modulator (Bertram et al., 2008). Among all APOE isoforms, APOE4 has been shown to exacerbate tau-mediated neurodegeneration, while the absence of APOE is protective in Patients with AD (Liu et al., 2013). Patients who carry at least one APOEε4 allele shows faster disease progression, and increased brain atrophy compared to non-APOEε4 carriers (LaDu et al., 1995; Shi et al., 2017). As previously mentioned, it inhibits the gene that produces SirT1, a molecule that has been associated with longer lifespans and has anti-Alzheimer’s properties and instead It’s linked to nuclear factor kappa B (NF-κB) activation, which encourages inflammation (Shi et al., 2017). This explains why ApoE4 is linked to an elevated inflammatory response: it suppresses multiple genes that inhibit inflammation while accelerating the NF-κB that stimulates it (Teng et al., 2017).

Aβ binding to APOE and other apolipoproteins was tested in different *in vitro* (Shi et al., 2017; Zhang et al., 2021). Even though the binding was consistently verified, none of those investigations suggested that variations in APOE-Aβ binding were linked to an increased risk of AD (Keren-Shaul et al., 2017). According to Yuan et al., TREM2 deficiency increased the amount of diffuse amyloid plaques that covered a greater surface area due to longer and more branched amyloid fibrils (Yuan et al., 2016a). Through TREM2 binding APOE evaluates the phagocytosis and APOE-Aβ uptake, while the TREM2 R47H variant has less affinity to bind with APOE (Tao et al., 2018; Sheng et al., 2019). Due to its dysregulation of neuroinflammation and elevation of AD risk, the missense mutation R47H of TREM2 is linked to AD risk (Ruganzu et al., 2021). A dose-dependent reduction in TREM2 inhibits the accumulation of myeloid cells surrounding Aβ plaques. In addition, plaque number and size are decreased in TREM2 deficiency (Wang et al., 2016; Yeh et al., 2016).

Microglia in plaque-loaded brain areas of AD transgenic mice expressed more TREM2, suggesting a significant role for TREM2 against AD (Yuan et al., 2016a). Growing data indicates that TREM2 deficiency support microglial phagocytosis and maintain microglial responses to Aβ deposition through inhibit the transition of microglia from a homeostatic to a disease-oriented state (Wang et al., 2016; Yuan et al., 2016b). TREM2 in blood and CSF can act as biomarker for the diagnosis of early AD since, the TREM2 levels in CSF increase in the early stages of AD, while it decreases in late stages (Wang et al., 2016).

Beside the two last popular AD hallmark genes, recent data suggests that the fractalkine ligand and its microglial receptor (CX3CL1/CX3CR1) can influence pathologies related to tau by controlling microglial migration and attracting monocytes to the brain (Lyons et al., 2009; Joaquín Merino et al., 2016).

Microglia most likely proliferate more quickly and assemble around fibrillar amyloid plaques because of dysregulated fractalkine/CX3CR1 signaling, brought on by CX3CR1 receptor deletion, indicating that CX3CR1 has been found to maintain microglia in an inactive, non-neurotoxic condition (Lyons et al., 2009; Bhaskar et al., 2010; Lee et al., 2010).

Mice deficient in CX3CR1 showed a alters the inflammatory milieu, decreased neuronal loss, and increase of the amount of Aβ phagocytosis mediated by microglia however an aggravated tau phosphorylation was also detected (Yin et al., 2017).

Similarly, colony-stimulating factor 1 receptor (CSF1R), inhibition has attenuated the neurodegeneration process caused by tau proteins (Chadarevian et al., 2023). Mutation of IFNγ receptors increases Aβ synthesis and microglial activation (Orihuela et al., 2016; Huang et al., 2018).

The CSF-1-CSF-1R pathway, which is mainly active in reactive microgliosis conditions has also been connected to microglia survival in the context of TREM2 expression (Öst et al., 2006; Spangenberg et al., 2019). This pathway affects Aβ clearance. A similar mechanism may also be involved in microglial survival, as it has been shown that TREM2 promote macrophage survival via CSF-1R pathway (Chadarevian et al., 2023). The role of CSF-1R signaling in microglia survival is detected by a study indicating that TREM2-deficient microglia to exhibit reduced survival at low CSF-1 concentrations (Mancuso et al., 2019).

The genetic deletion of the inflammatory NLR family pyrin domain containing 3 (NLRP3) facilitates the synthesis of IL-1β and improves Aβ clearance by microglia as well as cognitive function in AD mice (Wang Z. et al., 2020; Bai and Zhang, 2021). NLRP3 activation increase the AD pathogenesis by damaging the microglia mitochondrial aggregation and impairs the structural and functional integrity of mitochondria by increasing the release of proinflammatory cytokines (Liang et al., 2022). All the genes related to microglia activity are listed in Table 1.

**Table 1:** Summarizes all the genes related to microglial activity and their functions in AD.

Gene	Function	Expression	References
**Microglia genes in Aβ pathogenesis**			
SR-A	Regulation of microglia phagocytosis	Increased in AD	Frenkel et al. (2013)
CD36	Regulation of microglia phagocytosis	Increased in AD	Kim et al. (2017)
RAGE	Regulation of microglia phagocytosis	Increased in AD	Deane et al. (2012)
*APOE*	Regulation of microglia phagocytosis	Increased in AD	Nguyen et al. (2020)
*CR1*	Modulate microglia phagocytosis of Aβ	Increased in AD	Crehan et al. (2013)
*CD33*	Modulate microglia phagocytosis of Aβ	Increased in AD	Griciuc et al. (2013)
*TREM2*	Modulate Aβ phagocytosis	Decreased in AD	Ruganzu et al. (2021)
*ABCA7*	Modulate microglia phagocytosis of Aβ	Increased in AD	Aikawa et al. (2019)
**Microglia genes in Neuroinflammation**			
NLRP3	Modulate microglia-mediated inflammatory response	Increased in AD	Heneka et al. (2013)
BACE1	Increasing inflammatory responses	Increase in AD	Singh et al. (2022)
SOCS	Regulate the balancing of inflammatory response	Decreased in AD	Ruganzu et al. (2021)
SHIP1	Modulate microglia-mediated inflammatory response	Decreased in AD	Terzioglu and Young-Pearse (2023)
CX3CR1	Regulate *tau phosphorylation*	Decreased in AD	Cho et al. (2011)
**Microglia genes in tau pathology**			
CSF1R	Modifying tau-mediated neurodegeneration	Increased in AD	Spangenberg et al. (2019)
APOE	Modifying tau-mediated neurodegeneration	Increased in AD	Shi et al. (2017) and Nguyen et al. (2020)
TREM2	Regulating Aβ plaque and tau aggregates	Decreased in AD	Cheng et al. (2018)

## The blood–brain barrier and AD

The vascular blood–brain barrier (BBB), which serves as the brain’s primary interface with the outside world, is vital to maintaining brain homeostasis, it regulates the entry and exit of biological substances and is essential for shielding the brain parenchyma from blood-borne pathogens or exogenous substances into the central nervous system (Takechi et al., 2017). The BBB is composed of both molecular (the glycocalyx and basement membrane, junction complex) and cellular components (endothelial cells, pericytes, and astrocytes), The brain microvascular endothelial cells have developed a junction complex such as tight junction which sounds to be a very early feature of BBB development, separating blood from CNS by brain endothelial cells and provide the best conditions for synaptic and neural activity by certain ion channels and variety of efflux transporters (Halliday et al., 2000; Du et al., 2018; Khan et al., 2023). Under normal conditions, the BBB is relatively impermeable, the disruption of BBB and vascular dysfunction by the release of Many vasoactive substances, cytokines, and chemical mediators including glutamate, aspartate, taurine, ATP, endothelin-1, NO, TNF-α, and macrophage-inflammatory protein 2 (MIP2). Bradykinin, 5HT, histamine, thrombin, UTP, UMP, substance P, quinolinic acid, platelet-activating factor, and free radicals under pathologic circumstances such as AD have been associated with multiple molecular changes result in increased BBB permeability (Kadry et al., 2020).

Growing body of research indicates that BBB disruption is an early indicator of neurodegeneration, including AD.

Considering the major role of BBB to clear around 85% of AD-related forms of Aβ from the brain, BBB breakdown can dysregulate efflux and influx of Aβ transporters result in Aβ accumulation and decrease tight junction protein expression, which causes a greater influx of peripheral immune cells into the brain and capillary degeneration (Winkler et al., 2015).

**Figure 1:**
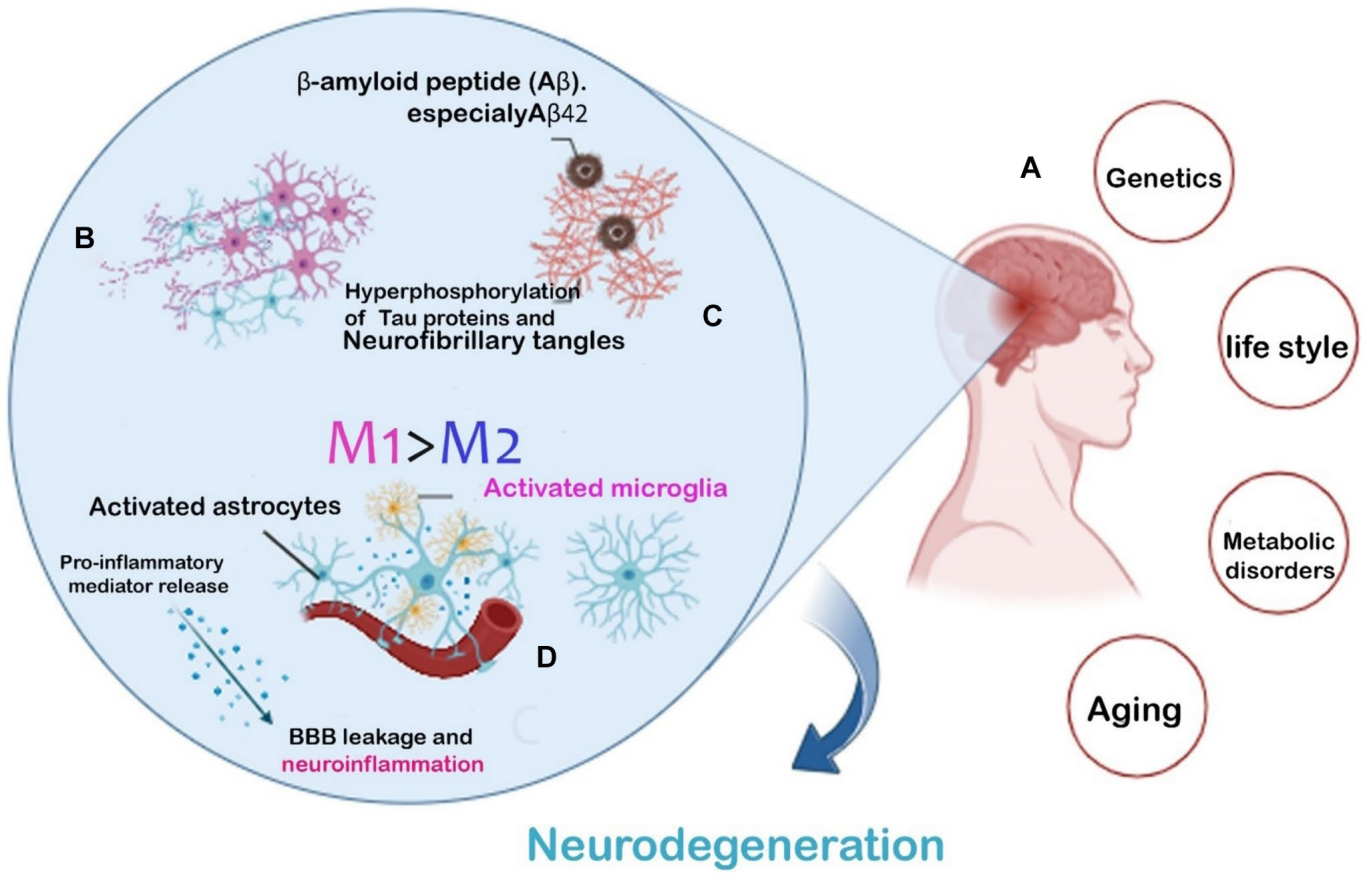
AD hallmarks and risk factors leading to neurodegeneration. Common risk factors leading to Two AD **(A)**. The main pathogenic hallmarks of AD are the extra-accumulation of amyloid-β plaques and Tau phosphorylation **(B)**. Microglial phenotype modification accelerating the neuroinflammatory response **(C)**. Inflammatory cytokines released from activated microglia causing BBB leakage and neurodegeneration **(D)**.

Clinical researches have demonstrated that decrease in pericyte quantity and coverage in the cortex and hippocampus of AD patients and mouse might be a reason for breaking BBB integrity through reducing brain microcirculation (Sweeney et al., 2018). Therefore, in AD patients numerous circulating soluble inflammatory mediators may impact on BBB malfunction specially during systemic inflammation and/or infection. Which is demonstrated by the fact that serum from mice treated with lipopolysaccharide (LPS) weakened the integrity of an *in vitro* BBB model more than serum from mice treated with a vehicle. Also aging can cause alterations in BBB as well as the immune system’s reactions. Aging cells usually take on a senescence-associated secretory phenotype which is associated with a transcriptional program that promotes Immune cells activation, migration, and infiltration by producing growth factors, cytokines, chemokines, and extracellular matrix proteases affect the BBB integrity (Lasry and Ben-Neriah, 2015; Figure 1).

## Inflammation and AD

Different clinical studies indicate the role of inflammation in cognitive decline especially in AD pathogenesis. Currently, inflammation is considered the third main hallmark of AD besides the hyper-phosphorylated tau protein and amyloid-beta (Aβ) protein accumulation (Bhaskar et al., 2010; Das and Ganesh, 2023). The molecules responsible for inflammation can be generally divided into cytokines and transcription factors (Šimić et al., 2016). Although the inflammatory response can be beneficial via accelerating the Aβ clearance, at the same time they can increase the Aβ and tau production, and promote neurodegeneration and synapse loss (Šimić et al., 2016).

**Figure 2:**
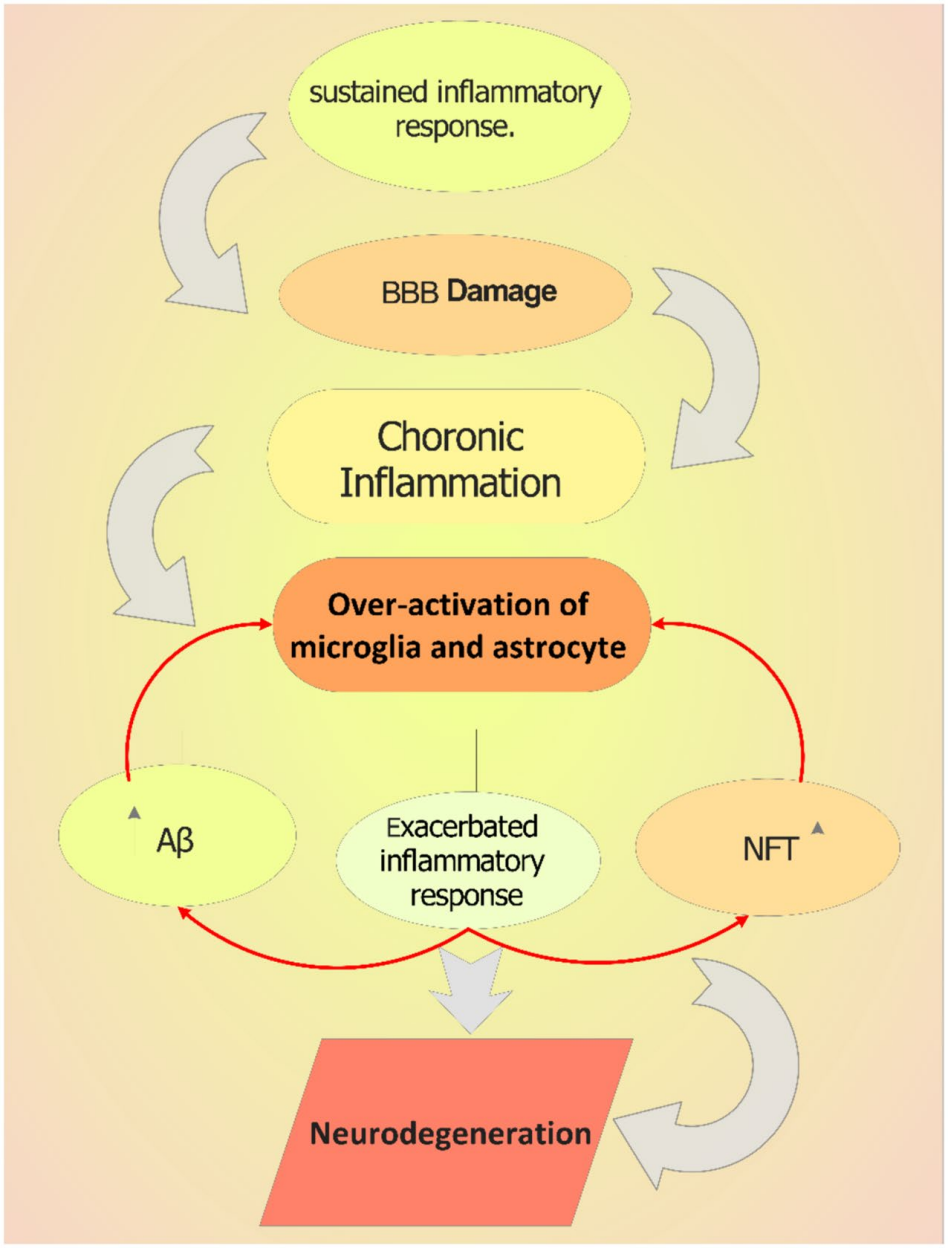
The role of chronic inflammation in AD pathology. Sustained inflammatory response can cause blood–brain barrier (BBB) damage, which increases the entrance and activity of other immune cells in brain. This over-activates microglia in the brain and triggers them to produce more inflammatory mediators including cytokines, which increase the extracellular plaques accumulation triggering neuroinflammation.

The balance between initiation and termination of immune response ensures the prompt removal of invasive pathogens and the cessation of excessive response within the central nervous system. This is crucial for the prevention of many diseases including the (Zheng et al., 2016). The inappropriate activation of inflammatory cytokines may lead to long-lasting alteration of regulatory neural gene expression. For instance, cytokines by interacting with different immune molecule groups such as the major histocompatibility complex class I (MHC I) can adversely affect the synaptic plasticity necessary for synapse formation and activity-dependent synaptic pruning (Ljunggren and Anderson, 1998). It is believed that these changes in synaptogenesis are fundamental to the causes of dementia. Additionally, cytokines can strongly stimulate the hypothalamic-pituitary-adrenal (HPA) axis, and increase the hormones release (Brosseron et al., 2014).Pro-inflammatory cytokines that cause chronic inflammation, like TNF-α, IL-6, and IL-1β, can influence and penetrate the blood–brain barrier (BBB), causing it to release proinflammatory mediators and increasing cell permeability, which permits leukocytes to enter the brain (Szczepanik et al., 2001; Swardfager et al., 2010). While anti-inflammatory cytokines are also produced. These include IL-1 receptor antagonist, IL-4, IL-10, and IL-11. These cytokines may be a part of a complex mechanism that prevents excessive neuroinflammation (Pousset et al., 2001; Guillot-Sestier et al., 2015). Activating the NF-κB pathway in microglia, can subsequently increase the amount of tau seeding and spreading and most AD patients are detected with considerably higher levels of NF-κB (Kaltschmidt et al., 1997). The silencing of microglial NF-κB cognitive abnormalities and homeostatic were restored. Hence, inhibiting the NF-κB pathway may offer a therapeutic approach to lessen AD pathogenesis (Sun et al., 2022). Finally, the other factor that can directly or indirectly increase inflammation and neuroinflammatory mediators is the overproduction of neutrophil extracellular traps (NETs) that induce macrophage activation and tissue damage (Brosseron et al., 2014; Swanson et al., 2018). Therefore, as shown in Figure 2, the permanent activation of astrocytes and Microglia can cause chronic inflammation. Chronic inflammation can be also caused by specific environmental factors, bacterial and viral infections, and Aging (Zhao et al., 2021). In chronic inflammation there is a major change in inflammatory pathway activation, leading to different immune responses and excessive production of inflammatory cytokines which lead to neuroinflammation (Figure 2; Neurath and Finotto, 2011).

## Inflammatory biomarkers and AD

Currently, Aβ42and phosphorylated tau proteins are the main fluid-based biomarkers of Cerebrospinal fluid (CSF) in clinical practice (Bălaşa et al., 2020). However, there are still limitations in their specific detection based on their low concentration in blood (Noble et al., 2014; Galizzi and Di Carlo, 2023). As mentioned, inflammation plays a major role in AD development and among all different neuroinflammatory biomarkers which can be considered as therapeutic targets for drug design, cytokines, chemokines and transcription factors for their precise roles in the various stages of AD, possible medical applications, and easy isolation from blood or CSF have attracted a lot of attention (Zheng et al., 2016; AmeliMojarad et al., 2022; Park et al., 2022).

Different research groups indicated the cytokine levels alternation in AD patients. For example, IL-1β, TNF-α, NF-κB and chemokines like CCL2 has found to be increasing in AD patients which can also be used as inflammatory markers (Bălaşa et al., 2020).

Fast-progressing AD is linked to IFN-γ polymorphism implies that this cytokine may actively contribute to accelerating the progression of AD specially the LOAD (O’Bryant et al., 2017).

Dysregulation of the cytokines and chemokines can cause neuroinflammatory modulation, altering the microglia phenotype, and reducing microgliosis which accelerate the AD progression (Swanson et al., 2018; Zhang et al., 2021). Nonetheless, the most recent meta-analysis revealed substantial heterogeneity in certain comparisons but no significant differences in cytokines, such as IL-1β, IL-6, IL-8, IL-10, or TNF-α, were discovered between AD patients and healthy controls (Blennow and Hampel, 2003; Newcombe et al., 2018).

Other inflammatory biomarkers in Alzheimer’s disease may include IL-33 and the soluble form of its receptor ST2 (sST2). In animal models of Alzheimer’s disease, IL-33 stimulates microglia and protects against Aβ plaques, despite its association with inflammation (Fu et al., 2016).

A 1-year follow-up study indicated that MCI and AD patients with positive IL-33 expression in serum performed better on cognitive tests, adding to the evidence for IL-33’s benefit. The explanation for the increase in IL-33 in AD and MCI patients’ plasma is surprising, given higher levels of this cytokine have been related to improved cognitive function. Recent research suggests that higher levels of sST2 in AD patients buffer the physiological effects of IL-33 and may play a role in the cognitive function impairment associated with AD (Fu et al., 2016; Liang et al., 2020). Moreover, based on the damaged blood–brain barrier (BBB), different proteins can pass through BBB therefore, the blood of AD patients can reflect the AD progression-related targets. More importantly, the large surface area of the blood–brain barrier can be considered as potential for therapeutic intervention (Sweeney et al., 2018; Niculescu et al., 2020).

Therefore, detecting the well-established inflammatory biomarkers and methods for early diagnosis and monitoring of AD patients can be considered as alternative method of AD identification. However, cytokines may not be sufficient to demonstrate that an imbalance in cytokine levels is the sole cause of AD based on their overlapping with other neurodegenerative disease and aging. Therefore, it makes more sense to combine the use of several proteins given the unpredictable results of using a single cytokine level.

But few sets of biomarkers have demonstrated consistent performance and good reproducibility since the first AD prediction model comprising 18 plasma biomarkers with multiple cytokines was proposed. Using hypersensitive methods, such as immunoprecipitation-mass spectrometry (IP-MS), and single-molecular mass analysis (SIMOA) can detect the minor changes in the Aβ plasma level in patients with AD (Wu et al., 2021; Nijakowski et al., 2024). A more sensible strategy is to use multiple proteins in combination (Ray et al., 2007; Zheng et al., 2016). However, only a small number of biomarker sets have demonstrated consistent performance and good reproducibility since the first AD prediction model comprising 18 plasma biomarkers with multiple cytokines was proposed (Ray et al., 2007). Furthermore, a combination of soluble IL-6 receptor (sIL-6R), tissue inhibitor of metalloproteinases-1 (TIMP-1), and soluble TNF-α receptor I (sTNFR-I) in CSF was found to provide the best prediction to AD among other molecules after screening 120 inflammatory molecules in CSF and serum of AD, MCI, and healthy controls using protein-array analysis (Richens et al., 2014; Delaby et al., 2015). Future research on AD should look at pathogens other than Aβ and examine how cytokines interact with other players. New genes and proteins can only be discovered through the creation of brain banks, while genome-wide association studies and online database analysis will continually update polymorphism information linked to AD (Delaby et al., 2015; Khan and Alkon, 2015). Table 2 summarized the recent neuroinflammatory biomarkers related with AD.

**Table 2:** List of neuroinflammatory biomarkers for AD.

Inflammatory markers	Type	Function in inflammation	References
IL-1α and IL-1β	Proinflammatory cytokines	Increased in CSF of AD patients	Forlenza et al. (2010)
ICAM-1	Adhesion molecule	Increased in CSF of AD patients	Rentzos et al. (2004)
VCAM-1	Adhesion molecule-1	Increased in CSF of AD patients	Borradaile and Pickering (2009) and Dou et al. (2013)
TNF-α	Proinflammatory cytokines	Increased in serum and CSF of AD patients	Frankola et al. (2011)
IL-6	Proinflammatory cytokines	Increased in serum and CSF of AD patients	Halliday et al. (2000)
IL-12	Proinflammatory cytokines	Increased in serum and CSF of AD patients	Zhang et al. (2023)
NF-κB	Transcription factor	Transcription factor that activates genes related to inflammation	Ju Hwang et al. (2019) and Liu et al. (2021)
CCL2	Chemokines	Increased in serum and CSF of AD patients	Westin et al. (2012)
IL-8	Pro-inflammatory	Increased in serum and CSF of AD patients	Galimberti et al. (2003)
IL-33	Pro-inflammatory	Increased in plasma of AD patients	Fu et al. (2016) and Liang et al. (2020b)
Progranulin	A growth factor	Increased in AD patients plasma with a potential for early prediction of AD patients	Kanazawa et al. (2016)
YKL-40	Mammalian chitinase-like proteins	Increased in AD patients’ plasma/serum increasing the neuroinflammation in astrocytes	Vergallo et al. (2020) and Zhang et al. (2023)

## Therapeutic strategies for AD

Novel therapeutics are being offered by the recently made connections between inflammation and neurodegeneration (Wu et al., 2021). Currently, a major treatment strategy for AD is the reduction of toxic Aβ plaque accumulation and generation and reducing the inflammatory responses (Muralidar et al., 2020; Wang Z. et al., 2020). Even though there is still no known treatment for AD, NSAIDs are commonly used drugs for AD with the ability to decrease of Aβ plaque load, microglial activation, and proinflammatory cytokine levels. Currently the most promising drugs in reducing inflammation are COX-2 inhibitors Celecoxib and roficoxib which attenuate the neuroinflammation in AD (Moride et al., 2003; Miguel-Álvarez et al., 2015).

COX-2 inhibitors work by inhibiting the cyclooxygenase (COX-1 and COX-2 enzyme), arachidonic acid cannot be converted into prostaglandins, or prostacyclin without cyclooxygenase which have degenerative effect. And can raise Aβ levels (Moride et al., 2003; Benito-León et al., 2019).

It’s interesting to note that degenerative brain cells express high levels of COX-2; therefore, blocking COX may lessen AD. Aβ-induced microglial activation may occur directly or indirectly, leading to an increase in COX-2 which can be found during inflammation (Moride et al., 2003). Compared to control brains, AD brains exhibit higher levels of COX-1 and COX-2 (Moussa and Dayoub, 2023).

Research using animal models of AD has demonstrated the potential benefit of NSAIDs against AD. For instance, oral administration of ibuprofen, a nonspecific COX inhibitor, at the outset of amyloid plaque formation in transgenic mice overexpressing APP reduced glial activation and plaque density (Moussa and Dayoub, 2023). In a different experiment, treated rats with indomethacin, reduced microglial activation, improved the hippocampus over time, and avoided working memory problems. Furthermore, and elevated COX-2 levels were generated in mice given an intracerebroventricular injection of Aβ (Karkhah et al., 2021). In addition, pretreatment with the specific COX-2 inhibitor NS398 reduced COX-2 levels and cognitive impairment (Minter et al., 2003). Further studies have demonstrated that therapy with ibuprofen and naproxen in transgenic mice models of AD Other studies of NSAIDs in human cell cultures have raised hopes for its usage in AD treatment (Wilkinson et al., 2012; Linda and Hershey, 2019; Steven Karceski, 2019). For instance, the overexpress APP695NL, in human neuroglioma cells identified different NSAIDs that can selectively reduce Aβ42 such as sulindac, ibuprofen, and diclofenac (Weggen et al., 2003).

Activating PPARγ, a transcriptional factor that suppresses the expression of proinflammatory genes by blocking the activity of other transcription factors like NFκB, AP-1, and STAT1, is another potential neuroprotective mechanism of NSAIDs. Additionally, proinflammatory genes can be suppressed by PPARγ in the vasculature and myeloid lineage cells like macrophages and microglia (Daynes and Jones, 2002; Heneka et al., 2011).

Consequently, pioglitazone, a PPARγ agonist, has been used in clinical AD research suppressing the expression of genes that promote inflammation to regulate transcription (Geldmacher et al., 2011).

However, NSAID usage is only beneficial in the early stages of AD, because, with the start of the Aβ deposition process, NSAIDs are ineffective and even dangerous because they decrease microglial inflammation, which mediates the clearance of A despite its negative effects (Ho et al., 2006). Targeting NLRP3 inflammasome of microglia is another strategy against AD and AD-related inflammatory responses, a small molecule NLRP3 inhibitor such as JC-124, and MCC950 has been discovered which vigorously pro-inflammatory cytokines, chemokines, and ROS in AD however, along with more comprehensive evaluations of the outcomes, could produce delightfully unexpected results (Yin et al., 2018; Kelley et al., 2019; Zhang et al., 2021; Sharma et al., 2023). Minocycline is a tetracycline with anti-inflammatory qualities that can cross the blood–brain barrier (BBB) (Garcez et al., 2019). An *in vivo* study suggests that minocycline reduces Aβ accumulation and attenuates microglial activation because it inhibits the NLRP3 inflammasome (Li et al., 2016; Garcez et al., 2019).

Nicodipine (P2X7R antagonists), a dihydropyridine calcium channel antagonist, has also been shown to confer neuroprotective effects by reducing the levels of activated NF-κB and inhibiting the release of mature IL-1β in Aβ-stimulated microglia (whose potential target is P2X7R), which plays a permissive role in NLRP3 inflammasome activation and cytokines release (Ryu and McLarnon, 2008; Di Virgilio et al., 2017; Huang et al., 2023). The list of recent agents for treatment strategy of AD is provided in Table 3.

**Table 3:** Current agents for therapeutic strategies in AD.

Drug	Targets	Function	References
Minocycline	NLRP3 inflammasome blockage	Attenuates microglial activation and reduces Aβ accumulation	Li et al. (2016)
MCC950	NLRP3 inflammasome blockage	Attenuates microglial activation and reduces Aβ accumulation	Jiao et al. (2020)
JC-124	NLRP3 inflammasome blockage	Attenuates microglial activation and reduces Aβ accumulation	Yin et al. (2018) and O’Brien et al. (2020)
Ibuprofen	NLRP3 inflammasome blockage	Attenuates microglial activation and reduces Aβ accumulation	Wilkinson et al. (2012)
Edaravone	NLRP3 inflammasome blockage	Attenuates microglial activation and reduces Aβ accumulation	Yang et al. (2015)
P2X7Rinhibitor	NLRP3 inflammasome blockage	Attenuates microglial activation and reduces Aβ accumulation	Huang et al. (2023)
P22	CD33 inhibitor	Increased Aβ phagocytosis	Boulanger (2009)
Lintuzumab	CD33 inhibitor	Increased Aβ phagocytosis	Miles et al. (2019)
4D9 antibody	TREM2 Modulator	Boosting microglial phagocytosis	Wang et al. (2016)
AL002c	TREM2 Modulator	Neuroprotective effects via reducing Aβ	Wang S. et al. (2020)
AL002a	TREM2 Modulators	Neuroprotective effects via reducing Aβ	Cheng et al. (2018)
PLX3397	CSF1R inhibitor	Suppress tau propagation	Sosna et al. (2018)
PLX5622	CSF1R inhibitor	Prevent plaque formation	Spangenberg et al. (2019)

## Conclusion

Chronic inflammation is the third core pathology in the progression of Alzheimer’s disease, alongside the well-known activities of Aβ and tau. Microglia play a crucial part in this process, activated microglia are thought to be the primary source of pro-inflammatory mediators released, such as cytokines, which drive inflammatory cascades in the CNS, resulting in neuroinflammatory modulation. Activated microglia can also enhance blood–brain barrier (BBB) permeability, synaptic loss, and neurodegeneration in the brain, accelerating the AD pathogenesis. Since there are still no effective therapies in terms of disease attenuation or prevention, further research is needed to unrevealing the potential reliable biomarkers for monitoring AD in early stages (Leng and Edison, 2020).

Inflammatory markers alternation in patients with AD can be considered as a new means to track AD progression. Novel biomarkers related to neuroinflammation such as proinflammatory cytokines and chemokines are mainly altered in in patients with AD. However, there are still limitation for considering proinflammatory markers as AD specific biomarkers, since many neurodegenerative diseases have similar clinical presentations, it is possible that their changes be explained by aging or other systemic disease. However, based on their easy extraction and interpretation, they can still be considered the best first-step biomarkers in the multi-step AD process. As a result, we can improve the accuracy of AD diagnosis and treatment plans in the near future by using the multiplex model, which combines various blood markers and proteins of AD patients.

## Author contributions

MaA: Conceptualization, Data curation, Writing – review & editing. MeA: Visualization, Writing – original draft, Writing – review & editing. XC: Funding acquisition, Supervision, Writing – review & editing.
